# Prevention and Management of Multimorbidity in Southeast Asia: A Narrative Review

**DOI:** 10.1002/puh2.218

**Published:** 2024-07-15

**Authors:** Xiyu Feng, Haribondhu Sarma, Matthew Kelly

**Affiliations:** ^1^ Department of Applied Epidemiology National Centre of Epidemiology and Population Health The Australian National University Canberra Australia

**Keywords:** controlling, intervention, management, multimorbidity, multiple chronic conditions, non‐communicable diseases, prevention

## Abstract

Multimorbidity, the coexistence of two or more chronic conditions, presents a growing global challenge, particularly in low‐ and middle‐income countries such as Southeast Asia. This trend necessitates the development of sustainable integrated care models to prevent and manage multimorbidity effectively. However, progress in this area has been hampered, especially in underdeveloped regions, by various barriers, including the epidemiology of multimorbidity, how to get different specialists and doctors to work together most availably and manage the multiple medication issues and how to develop cost‐effective approaches to reduce the health burden of multimorbidity. Preventive measures in Southeast Asia, which could tackle multiple components which commonly comprise multimorbidity, include enhancing health literacy and health promotion through school‐ and community‐based educational activities, primary healthcare and related policies on employing taxes on tobacco, alcohol and sugary beverages. The social determinants of health‐encompassing poverty and low education may also influence research on multimorbidity. Moreover, stakeholder engagements involving national governments, World Health Organization (WHO) and Association of Southeast Asian Nations (ASEAN) are crucial. Management strategies focus on integrated care models, including patient‐centred primary healthcare, digital healthcare technologies, and medication management to control polypharmacy. Although research on multimorbidity in Southeast Asia is increasing, translating findings into practical measures was limited. Future efforts should prioritize evidence‐based approaches to prevent and manage multimorbidity effectively, addressing challenges like health system focusing on single chronic disease treatment independently, resource limitations, healthcare provider shortages and individual adherence issues. These ways promise to enhance the quality of life and health outcomes in this region.

## Graphical abstract

In Southeast Asia, preventing and managing multimorbidity require enhancing health literacy, addressing poverty and low education, stakeholder collaboration and integrated care models, while overcoming resource and system challenges to improve health outcomes and quality of life.

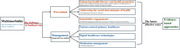

## 1 Introduction

Multimorbidity is a condition in which a person suffers from two or more chronic diseases at the same time [1, 2, 3]. One of the most important drivers of multimorbidity prevalence is population ageing, and older people are more susceptible to multimorbidity [1, 4, 5]. Multimorbidity has become an increasingly recognized global problem, as it complicates treatment regimens, increases healthcare utilization and poses significant burdens on individuals and healthcare systems [1, 4].

Research on effective prevention and management approaches for multimorbidity is lacking, especially in low‐ and middle‐income countries (LMICs), where the focus has primarily been on individual non‐communicable diseases (NCDs) [1, 5]. LMICs generally have younger populations and lower multimorbidity prevalence. However, rapid demographic shifts in recent decades, particularly in Southeast Asia, have led to population ageing and a transition from infectious to chronic diseases as major health burdens [1, 6, 7]. Additionally, LMICs often face challenges such as poorly resourced healthcare systems, outdated medical technologies and uneven access to healthcare services, which can delay diagnosis and contribute to the development of multimorbidity [1, 4, 5].

The prevention and management of multimorbidity is not simply a matter of considering NCDs but also including attention to chronic infectious diseases such as tuberculosis (TB) and HIV, and psychiatric disorders, including bipolar disorder and depression [1, 4, 6]. A particular challenge of multimorbidity is the associated use of multiple medications (polypharmacy) and increased risk of drug interactions. This is especially challenging for primary healthcare in low‐resource settings, which may be the first point of contact between the healthcare system and the patient. Multimorbidity may require a higher level of healthcare demand [1, 2] in LMICs settings where primary healthcare systems may not be adequately resourced [1, 2, 3].

Preventing and managing multimorbidity present a complex challenge that demands a comprehensive approach [1, 2, 3, 4], considering various risk factors, including individual, social and environmental determinants. However, prevention and management approaches for multimorbidity remain limited, with most strategies focusing on individual chronic conditions [1, 5, 6]. Managing multimorbidity requires an integrated approach that acknowledges the intricate interactions between multiple health conditions, treatments and patient preferences [4, 5]. It is crucial to optimize disease control while minimizing treatment burden and the potential harms of polypharmacy [5, 6]. Emphasizing patient‐centred care, shared decision‐making and coordinated care across healthcare settings are essential for successful prevention and management of multimorbidity [1, 6].

In Southeast Asia, the rising prevalence of multimorbidity is driven by factors like population ageing, changing disease patterns and lifestyle shifts due to rapid urbanization and industrialization [1, 7, 8]. Unhealthy diets, physical inactivity and substance abuse are key contributors to multimorbidity risk [8, 9]. Additionally, the region is witnessing a significant increase in its elderly population, projected to reach 24.1% by 2050 from 8.8% in 2018 [8]. This demographic shift has led to a transition in health challenges, with a decline in communicable diseases like malaria and an increase in chronic NCDs such as cardiovascular diseases (CVD), diabetes and chronic respiratory diseases. Multimorbidity prevalence has risen from around 4.5% to approximately 10% in recent years, with hypertension, CVD and diabetes mellitus being the most prevalent chronic diseases [7, 9, 10]. Despite efforts such as universal health insurance schemes to tackle chronic diseases, integrated care for multimorbidity remains a challenge in Southeast Asian healthcare systems [7, 9, 10].

This article provides a comprehensive overview of the current status of multimorbidity prevention and management strategies in Southeast Asia. Through the exploration of prevention initiatives, covering health literacy promotion, addressing social determinants of health and stakeholder engagement, as well as methods for managing multimorbidity focusing on integrated care models, including strengthening primary healthcare, optimizing medication management and utilizing digital health tools, to offer insights and recommendations for guiding future efforts in multimorbidity prevention and management in the region.

## 2 Search Strategy

Articles were selected on the basis of inclusion criteria focused on the prevention and management of multimorbidity and/or NCDs in Southeast Asia. These criteria included studies conducted in Southeast Asian countries or the region defined by the Association of Southeast Asian Nations (ASEAN), with publication dates between 1990 and 5 April 2024, given the term ‘multimorbidity’ originated in the early 1990s [1]. Exclusion criteria encompassed conference papers, studies outside Southeast Asia and those not published in English within the specified timeframe.

Database searches on Google Scholar, PubMed and Scopus were conducted using terms related to exposure (prevention, management, or their related synonyms), outcomes (multimorbidity or it related synonyms) and location (Southeast Asia or specific countries). The searches were divided into prevention and management categories, with terms combined using ‘AND’ across categories and ‘OR’ within categories. Hand searches of retrieved study references were also performed to find additional relevant papers.

## 3 Prevention of Multimorbidity

**FIGURE 1:**
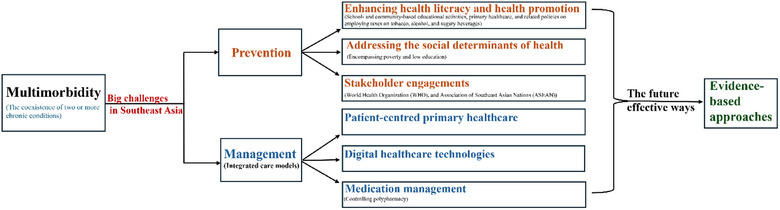
Key factors for the prevention and management of multimorbidity in Southeast Asia.

Many of the chronic diseases which comprise multimorbidity share common behavioural risk factors, including alcohol consumption and smoking, sedentariness and diets high in fat, sugar and sodium, socio‐economic risk factors, including low education and income, and health inequalities (inability to obtain adequate medical treatment and to afford the corresponding medical expenses) [1, 10]. Common approaches to preventing multimorbidity include improving health literacy and health promotion, addressing social determinants of health and the stakeholder engagement. The summary of key factors for the prevention and management of multimorbidity in Southeast Asia is shown in Figure 1.

### 3.1 Improvement of Health Literacy and Health Promotion

Health literacy efforts can raise awareness of risk factors associated with multimorbidity and the importance of adopting a healthy lifestyle. Promoting knowledge about preventive measures such as regular physical activity, healthy eating habits, smoking cessation and stress management can empower individuals to make informed choices and reduce the risk of developing multimorbidity [11]. For example, many countries, such as Singapore, Malaysia, Indonesia and Thailand in Southeast Asia, implement comprehensive health literacy programmes in schools that cover a wide range of topics, including nutrition, physical activity, mental health, sexual and reproductive health, and the prevention of chronic conditions [7, 9]. The curriculum aims to equip students with the knowledge and skills to make healthy choices and prevent NCDs [7, 9]. Although there is less clear policy on health literacy specifically to prevent multimorbidity, addressing common risk factors for NCDs through health literacy in a study conducted in Singapore reduced the prevalence of multimorbidity [12].

In addition to schools, health literacy in the community is also very important. Thailand and Malaysia encourage community‐based health education programmes facilitated by community health centres and community health volunteers [7, 9]. These programmes provide health education sessions, workshops and screenings to promote healthy behaviours and early detection of health issues [7, 9, 12].

As for the health promotion, lifestyles, including smoking and alcohol consumption, diets high in salt, sugar and oil, and sedentary behaviours, may cause or exacerbate multimorbidity. Vietnam, Myanmar and Thailand employ taxes on tobacco, alcohol and sugary beverages to curb consumption and manage health risks [7, 9, 13]. The Health Promotion Foundation of Thailand also advocates for community‐based physical activity programmes [13, 14]. Indonesia has implemented the ‘Gerakan Masyarakat Hidup Sehat’ (Healthy Living Community Movement) which promotes healthy lifestyles and NCDs and multimorbidity prevention through education, community engagement and policy changes such as tobacco control [15, 16]. Singapore's Health Promotion Board spearheads integrated strategies to promote healthy behaviours and prevent multimorbidity [17].

These health promotion activities in Southeast Asia are usually carried out through the preventative function of primary healthcare system. Primary healthcare in Southeast Asia is often the first point of contact for individuals seeking medical care [18, 19]. Reminding and urging people to start regular health check‐ups, screening programmes and risk assessments at a relatively young age can help to identify risk factors and potential health problems at an early stage so that multimorbidity can be prevented in a timely manner [18, 19]. Furthermore, primary healthcare providers can also be involved in health promotion and education activities to raise health literacy and awareness of healthy lifestyles and prevention of multimorbidity. These could include educating the population about the importance of a balanced diet, regular physical activity, smoking cessation and limiting alcohol intake [18, 19].

### 3.2 Addressing Social Determinants of Health

One way in which social determinants of health impact the risk of NCDs and multimorbidity is through socio‐economic status (SES). Lower SES limits access to quality healthcare and preventive services, leading to delayed or inadequate care and increased NCDs and multimorbidity risks [1, 18]. Limited economic resources often lead to unhealthy lifestyle behaviours such as poor nutrition, physical inactivity and tobacco use exacerbating these risks [18]. Housing conditions, air quality and neighbourhood safety, influenced by social determinants, directly affect health outcomes, especially for those with low SES [18]. Furthermore, high external stress levels among low SES individuals compromise their health further [18].

The social determinants of health drive health inequalities that contribute to the occurrence of multimorbidity. For instance, healthcare inequalities contribute to an increased prevalence of multimorbidity among low‐income people, as these individuals lack access to timely prevention and treatment related to multimorbidity [4, 5, 6]. Southeast Asian governments are addressing these determinants through policies establishing social protection mechanisms and universal health coverage [7, 9]. These policies aim to provide affordable, quality healthcare services to all citizens, regardless of SES, through initiatives like Universal Coverage Schemes (UCS) [7, 9].

It is vital to bridge cultural and ethnic disparities, particularly in Southeast Asia, where diverse languages and cultures exist. Language barriers can lead to miscommunication, delaying diagnosis and treatment, worsening health outcomes and contributing to multimorbidity [1, 6, 7, 9]. Cultural norms affect dietary habits, activity levels and mental health perceptions, impacting multimorbidity risk factors. Healthcare access disparities based on culture and SES worsen disease burdens in marginalized communities, increasing multimorbidity risks [1, 6, 7, 9].

Government initiatives such as Singapore's Community Health Assistance Scheme (CHAS) subsidize healthcare for low‐ and middle‐income individuals and marginalized groups, enhancing access to quality care [9, 19]. Although some Southeast Asian countries have social assistance programmes such as Indonesia's Family Hope Program, aiming to support marginalized groups, their impact on multimorbidity remains unclear [20]. Effective addressing of health determinants, including prevention of multiple diseases, relies heavily on government support and engagement [18, 21].

### 3.3 Stakeholder Engagement

With the epidemiological transition in the Southeast Asian region, the importance of NCDs has been increasing and all government efforts are required to address this challenge given the complex causal pathways. Governments in Southeast Asia have established inter‐ministerial committees or working groups involving health, social welfare, education and other related sectors to comprehensively address NCDs [7, 9]. For example, in Thailand, the Ministry of Interior and the Ministry of Health issued a joint statement on the prevention and control of NCDs [22]. Similar policies have been introduced in Indonesia [23] and Myanmar [24]. Although these measures aim to address some of the risk factors common across NCDs, little attention has been given specifically to multimorbidity.

The World Health Organization (WHO) and the ASEAN, the regional organization of 10 Southeast Asian and Pacific Rim countries, including Brunei, Cambodia, Indonesia, Laos, Malaysia, Myanmar, Philippines, Singapore, Thailand and Vietnam, whose governments cooperate to promote the socio‐cultural, economic and political progress of the region [25], have carried out a range of efforts to prevent NCDs such as *Implementation roadmap for accelerating the prevention and control of NCDs in South‐East Asia 2022–2030* [7] and *Framework for the Development and Implementation of Fiscal Measures on Sweet Beverages to Promote Health in ASEAN Member States* [25], but for multimorbidity prevention, these organizations do not have a dedicated programme.

This is also true for individual countries in the Southeast Asian region. In Indonesia, the government's project on chronic disease prevention in collaboration with community health centres (Puskesmas) does not address multimorbidity [26]. Moreover in Malaysia [27] and Singapore [28], the government's partnership with private healthcare providers and non‐governmental organizations (NGOs) to implement preventive healthcare programmes, including screening, health education as well as lifestyle interventions, only mentioned NCDs individually.

## 4 Management of Multimorbidity

Integrated care models play an important role in addressing the complex healthcare needs of individuals with multimorbidity. These models ensure that patients receive comprehensive and holistic treatment tailored to their specific combination of chronic conditions [29, 30]. The multimorbidity management could be optimized by integrated care models, emphasizing continuity of care, personalized interventions and efficient resource utilization. Consequently, they significantly contribute to improving patient outcomes and reducing healthcare costs associated with managing multimorbidity [29, 30, 31].

### 4.1 Enhancing Primary Healthcare

The crucial role primary healthcare plays in improving health literacy and prevention of multimorbidity, and this sector also plays a key role in the management of the conditions which make up multimorbidity [21]. Primary healthcare providers are trained to provide a person‐centred and making‐sharing continuum of care that addresses not only the physical health of the individual, but also their psychosocial and preventive needs [21, 29, 30].

Primary healthcare services are generally more accessible and affordable compared to specialized care. By managing multimorbidity at the primary healthcare level, individuals can receive timely and cost‐effective care [29, 30]. They are often not only the point of first screening and diagnosis for NCDs but also the treatment point. Although diagnosis, screening or acute care may occur in hospital settings, the primary healthcare sector is responsible for guidance on maintaining healthy lifestyles medication adherence, and general advice [29, 30]. Although the management of multimorbidity in Southeast Asia is still limited at this stage, with most of the management focusing on individual NCDs, they are also useful and instructive for multimorbidity [21, 29, 30].

The management of multimorbidity, like for individual NCDs, requires the development of well‐coordinated care models across different healthcare organizations and settings. Examples of such models can be found in Southeast Asian countries such as Thailand [31, 32] and Singapore [33, 34], where UCS programmes have been implemented. These programmes ensure the payment of medical expenses for the insured and promote the universalization and development of basic healthcare services [31, 32]. Through reasonable health insurance policies, medical institutions are encouraged to provide essential medical services and receive appropriate remuneration, making healthcare more accessible [31, 32]. As a result, people have better access to a range of healthcare services, from primary care to specialty and hospital services. Additionally, UCS can provide long‐term medical support to patients with chronic diseases and multimorbidity. For instance, in particular setting, it can incorporate routine blood pressure and blood glucose monitoring into medical consultations for hypertensive and diabetic patients, thus easing their financial burden [31, 32].

The management of multimorbidity in primary healthcare presents complexities beyond those of individual NCDs. An integrated care model incorporating patient‐centred care and decision‐sharing is recommended [35, 36]. The patient‐centred model prioritizes the patient's individual needs, culture and religious beliefs, treatment goals, values and preferences, recognizing their central role in healthcare decision‐making. Healthcare providers engage in open and honest communication, providing necessary information about conditions, treatment options, benefits and risks, involving patients in care planning and decision‐making [35, 36].

Multimorbid patients often encounter complex medical situations, including the simultaneous presence of multiple diseases, treatment regimen interactions and treatment choice uncertainty [37, 38]. For example, a patient dealing with both diabetes and hypertension may have unique treatment preferences and values, such as favouring non‐pharmacological interventions and cultural and religious taboos. A patient‐centred approach entails discussing these options openly, providing information on benefits and risks, and collaboratively developing a treatment plan aligned with the patient's preferences and values [37, 38]. This collaborative approach improves treatment adherence, satisfaction and overall effectiveness in managing multiple conditions [36, 38].

There are also many randomized controlled trials (RCT) on primary healthcare systems in the management of multimorbidity, especially in Europe and North America, and fewer studies in Southeast Asia [39, 40]. The management used in these RCTs includes self‐management, medication optimization and treatment refinement and generally involves intervention levels of patient‐level, provider‐level, organizational‐level and integrated level (combining two or three of the above levels). In terms of results, the integrated level has been found to be the most effective, although the results of the trials have been more mixed [39, 40]. Therefore, it can also be argued that integrated interventions are an important part of the future management of multimorbidity in primary care.

Thailand has prioritized primary healthcare in national reform efforts, employing a ‘task‐shifting’ strategy to train health workers for primary care [32]. Programmes, such as District Health Management Learning (DHML) and Contracting Unit of Primary Care (CUPC), focus on patient‐centred services for those with multimorbidity [41]. Similarly, Singapore has expanded community‐based primary care initiatives, like the National University Health System‐Regional Health System Integrated Interventions and Care Extension (NUHS‐RHS NICE programme), aiming to enhance post‐hospital discharge care for multimorbid patients [42]. Furthermore, in Indonesia, an integrated primary healthcare using a life course approach for managing multimorbidity is underway in this region, considering a variety of health determinants and socio‐economic factors such as consumption patterns, environment and physical activity [43].

Such integrated primary healthcare is based on the development of an individualized care plan that addresses disease management, medication management, lifestyle modifications and preventive measures based on the multimorbid patient's specific needs, goals and preferences and considers the patient's priorities, which are reviewed and adjusted on a regular basis in accordance with the patient's changing needs and preferences [1, 44]. This is undoubtedly a very effective way of managing multimorbidity, which is variable, and the only way to do so is through integrated, personalized and professional care [1, 44].

### 4.2 Polypharmacy and Medication Management

Medication management is also a significant aspect of control for multimorbidity. Concerns have been raised about the polypharmacy needs required to manage multiple conditions and the potential for interactions among different drugs. Drug combinations may increase adverse effects, and some medications reduce the efficacy of other medications [45, 46]. For example, antipsychotics may trigger diabetes and hyperlipidaemia and such drug may intervene the efficacy of hypoglycaemic drugs [45, 46].

Most Southeast Asian countries, such as Thailand, Myanmar and the Philippines, have the National List of Essential Medicines (NLEM), for treating common health problems that are safe and effective treatments [47, 48]. NLEM in these countries would offer healthcare providers with references and guidelines to determine appropriate medication use for various health conditions. It could help promote rational medication use by identifying essential medications that are safe, effective and cost‐efficient.

For patients with multimorbidity, the current NLEM in Southeast Asian countries may not fully meet their needs, as it primarily focuses on medications for single diseases. This can lead to challenges related to polypharmacy, where patients require multiple medications, increasing risk of drug–drug interactions, adverse drug events and drug‐related injuries [45, 46, 47, 48]. Thus, against this backdrop, efforts to reduce polypharmacy through trials, including those in Singapore [47], have become more common. The aim is to ensure patients receive a safe, rational and effective range of medications [49, 50].

### 4.3 Digital Health

In Southeast Asia, digital health is gaining importance particularly for remote communities [13]. Digital health technologies like telemedicine, mHealth and eHealth applications help to overcome the barriers to accessing healthcare services in Southeast Asia, especially in the primary healthcare sector. Telemedicine is a very important component of digital health, enabling patients to receive medical advice, consultation and follow‐up care without travelling or long waiting times [51]. In addition, digital health tools such as wearable devices, including real‐time blood pressure monitoring, home monitoring systems and mHealth apps that upload daily health data, are gaining acceptance in some countries in Southeast Asia [51].

Digital health can enable more efficient communication and record monitoring between specialists treating patients of multimorbidity [51, 52]. Patients can upload vital signs and medication data that are publicly available to all healthcare staff involved in the treatment, allowing these health professionals to discuss changes in their conditions together in real time and to remotely monitor their health status and intervene when necessary [52]. Moreover, it is convenient for these patients to be aware of their health changes without having to visit the healthcare provider's office [52].

Electronic health records (EHRs) are a very critical part of digital health platforms [51, 52]. EHRs serve as centralized repositories for storing and accessing comprehensive patient information. In the context of multimorbidity. EHRs provide healthcare providers with a comprehensive view of the patient's medical history, including diagnoses, medications, allergies and test results. This comprehensive information helps healthcare professionals understand the complexities and interactions among different conditions, enabling more accurate diagnoses and appropriate treatment plans [52, 53].

Thailand has been actively promoting the adoption of EHRs and has developed the Health Information System (HIS) to facilitate data sharing and integration among healthcare providers. This system includes initiatives aimed at improving chronic disease management, including multimorbidity [32, 54]. Thailand has implemented legal frameworks and guidelines to safeguard patient data privacy and security within the EHR system [54, 55]. Similarly, Singapore has made significant progress in EHR uptake and integration, with the implementation of the National EHR (NEHR) system. This platform offers healthcare providers a secure and comprehensive means of accessing patient information across various healthcare settings, supporting care coordination, medication reconciliation and evidence‐based decision‐making for multimorbidity management. Singapore also enforces robust regulations and privacy laws to protect patient data in electronic medical records [54, 56, 57]. Countries like Vietnam, Myanmar and Indonesia are also actively working on developing and delivering their own EHR systems [51].

## 5 Challenges of Prevention and Management of Multimorbidity

Limited healthcare resources in Southeast Asia, including infrastructure, personnel and funding, pose challenges for preventive services such as screenings and early detection [7, 9]. For instance, inadequate medical facilities and equipment hinder necessary screenings and early detection, particularly in remote areas with uneven healthcare distribution. Shortages of doctors and nurses lead to delayed or inadequate diagnostic and treatment services, impacting early intervention opportunities. Insufficient funding may result in healthcare workers lacking essential skills and knowledge, affecting service quality [7, 9, 22]. Overall, resource scarcity impedes the implementation of prevention and management strategies for multimorbidity [1, 19]. Fragmented healthcare systems exacerbate the issue, causing communication gaps and coordination issues among providers, potentially leading to treatment conflicts and errors.

Although digital health is gaining popularity in Southeast Asia, health information‐sharing and interoperability via digital platforms still encounter numerous challenges. Inconsistent standards, varying levels of digital health usage, and limitations in health information exchange infrastructure can hinder the sharing of patient information among healthcare providers [51]. This lack of interoperability affects coordination, continuity of care and a comprehensive understanding of a patient's multimorbidity [13, 58]. Patients with multimorbidity often have more complex medical data, and healthcare professionals may struggle to access all relevant information and identify the most critical health issues. Additionally, these individuals have complex medical needs, which may not be fully captured by existing EHRs [59].

Cultural beliefs, socio‐economic factors, and health‐seeking behaviours play a significant role in influencing multimorbidity prevention and management [39, 60]. Traditional practices, social norms and cultural beliefs can make an impact on the prevention and management of multimorbidity [1, 9, 60]. Southeast Asia is a diverse region with various ethnic groups, each with unique living habits, cultural practices and socio‐economic backgrounds. Understanding and addressing these cultural and socio‐economic factors are crucial for developing effective prevention and management strategies tailored to the local context. However, this aspect of multimorbidity prevention and management is still underexplored in Southeast Asia [1, 7, 61].

## 6 Recommendations

Although Southeast Asian countries have acknowledged the severity of multimorbidity and are actively addressing various issues related to it, there remains a pressing need to further implement and disseminate effective approaches for its prevention and management [9, 39]. It is essential that an evidence‐based approach should be paramount in guiding future research endeavours and policy development concerning multimorbidity prevention and management. Greater emphasis on implementing evidence‐based practices could improve the overall success in tackling the high burden of multimorbidity within the region [39, 61, 62].

As a first step, Southeast Asian countries may investigate the experiences of some high‐income countries (HIC) countries in the introduction of prevention and management policies that specifically target multimorbidity, rather than just single chronic conditions [1, 7, 9, 61]. The Australian government for example has developed a manual on multimorbidity prevention and management in 2021. The manual not only describes in detail the prevalence of multimorbidity but also mentions the threat factors as well as some means of prevention and treatment, providing very valuable information for controlling multimorbidity [61].

Promoting healthy lifestyles is crucial for preventing and managing in multimorbidity, particularly among lower SES individuals in Southeast Asia [1, 63, 64]. Social support, community linkage and policy advocacy are essential for addressing health inequalities and improving living conditions [1, 40, 65]. Health education programmes empower individuals to make informed health decisions, which could improve the outcome of multimorbidity [65]. Apart from taxation policies, providing resources and facilities for maintaining a healthy lifestyle is vital for effective multimorbidity management [66]. Strategies like increasing fruit and vegetable intake [67] and regular exercise [68] are effective in controlling multimorbidity.

Other areas that need further refinements are primary healthcare as prioritizing NCDs over infectious diseases is still relatively new in many countries in Southeast Asia. Efforts towards integrated care models and patient‐centred approaches require further refinement to effectively manage multimorbidity. Fragmentation and poor coordination within the primary healthcare system are common challenges, which can be addressed by promoting teamwork and communication among healthcare providers [7, 9].

Improving healthcare infrastructure, equipment and personnel is crucial, especially in remote areas of Southeast Asia. Constructing new medical facilities in these regions ensures access to basic services [1, 5]. Upgrading medical equipment enables advanced diagnostic and treatment capabilities. Providing multicultural training enhances staff skills to cater to diverse cultural backgrounds [1, 7, 9, 22]. Engaging community residents in facility management enhances service sustainability [1, 6, 7, 9]. These measures aim to enhance healthcare accessibility and quality, for better addressing the prevention and management of multimorbidity in Southeast Asia.

Besides providing patients with optimal medication regimens within a patient‐centred, shared decision‐making, integrated care model to solve the polypharmacy issues, it is essential to raise awareness of drug interactions, individual symptom monitoring and strengthen comprehensive care for patients with multimorbidity training of the team, including disease‐specific experts prescribing medications for multimorbid patients and involvement of pharmacists in reviewing medication lists to assess the risk of drug interactions [45, 46, 47, 48]. At the same time, lifestyle management and social support to reduce medication side effects would also be necessary, especially in the context of easy access to prescription drugs [54, 68, 69]. In Southeast Asia, there are many drugs such as antibiotics and painkillers that are available without prescription, which may also lead to drug abuse, especially in the background of multimorbidity [47]. Culturally sensitive prevention and management strategies are crucial in a multi‐ethnic region such as Southeast Asia to enhance the acceptability and effectiveness of interventions [1, 9, 60].

Although digital health in Southeast Asia is advancing, challenges persist with EHRs, especially in managing complex health needs of patients with multimorbidity. Current EHRs may struggle to capture timely changes in health status, and healthcare workers often face difficulty accessing comprehensive patient information due to varying standards [53, 54, 70]. To address these issues, enhancing EHR functionality, integrating them with other healthcare systems and standardizing practices are essential steps to cope with multimorbidity [52, 71].

## 7 Conclusion

Addressing multimorbidity in Southeast Asia requires comprehensive prevention and management strategies. Prevention entails enhancing health literacy, involving stakeholders and addressing social determinants of health. Management involves adopting integrated care models, covering prioritize patient‐centred primary healthcare, optimizing medication management and utilizing digital health technologies. Despite increasing research, translating findings into actionable interventions remains challenging. Future efforts should focus on evidence‐based decision‐making, strengthening healthcare infrastructure and developing relevant policies to overcome resource limitations and healthcare provider shortages. These endeavours aim to improve the quality of life and health outcomes for individuals in Southeast Asia.

## Author Contributions

**Xiyu Feng**: conceptualization, writing – original draft, writing – review and editing, data curation. **Haribondhu Sarma**: writing – review and editing, supervision. **Matthew Kelly**: writing – review and editing, supervision.

## Ethics Statement

An ethical statement is not applicable because this study did not involve human or animal research as it is a review article.

## Conflicts of Interest

The authors declare no conflicts of interest.

## Data Availability

Data sharing is not applicable to this article as no datasets were generated or analysed during the current study.
